# Lupus Cured by Large Quantities of Cod-liver Oil

**Published:** 1852-11

**Authors:** 


*Lupus cured by large quantities of Cod-liver Oil.*—L'Union Medicale relates, on the authority of another French Journal, a case of lupus which was admitted into the hospital of Ghent; the disease had attacked the face and chest, and was of long duration. The patient at first took half a pound of the oil twice a day; this quantity was gradually increased to three pounds daily, with occasional interruptions when it disagreed. Generous diet was at the same time allowed, and the ulcerated spots were touched with tincture iodine, lemon-juice and nitrate of silver. In about seven months the cure was complete. About 265 pounds of the oil had been taken.—*New York Medical Times.*

